# Epigenetic changes during sepsis: on your marks!

**DOI:** 10.1186/s13054-015-1068-5

**Published:** 2015-10-15

**Authors:** Aurélien Bataille, Pierre Galichon, Marie-Julia Ziliotis, Iman Sadia, Alexandre Hertig

**Affiliations:** Department of Anesthesia and Intensive Care, Groupe Hospitalier Universitaire Saint-Louis-Lariboisière-Fernand-Widal, 75010 Paris, France; Inserm UMR_S 1155, “Rare and common kidney diseases, matrix remodelling and tissue repair”, Hôpital Tenon, 75020 Paris, France; Urgences Néphrologiques et Transplantation Rénale, Hôpital Tenon, 75020 Paris, France

## Abstract

Epigenetics is the study of how cells, organs, and even individuals utilize their genes over specific periods of time, and under specific environmental constraints. Very importantly, epigenetics is now expanding into the field of medicine and hence should provide new information for the development of drugs. Bomsztyk and colleagues have detected major epigenetic changes occurring in several organs as early as 6 h after the onset of a mouse model of multiple organ dysfunction syndrome induced by *Staphylococcus aureus* lung injury. Decrease in mRNA of key genes involved in endothelial function was found to be associated with (and potentially explained by) a decrease in permissive histone marks, while repressive marks were unchanged. We discuss here the limitations of a whole-organ as opposed to a cell-specific approach, the nature of the controls that were chosen, and the pitfalls of histone modifications as a cause of the eventual phenotype. While the use of ‘epidrugs’ is definitely welcome in the clinic, how and when they will be used in sepsis-related multiple organ dysfunction will require further experimental studies.

## Commentary

Progress in the science of epigenetics has increased our understanding of the interaction between living eukaryotes and the environment. We read with great interest the article by Karol Bomsztyk *et al.*, recently published in *Critical Care* [1]. To our knowledge, this is the first time that the community of intensivists has been provided with translational research exploring a major aspect of epigenetics (here, histone modifications) in sepsis-related multiple organ dysfunction syndrome (MODS).

Epigenetics is a comparatively recent science, rapidly expanding in the field of medicine. Its definition has greatly evolved during the past century. At present it is defined as ‘the structural adaptation of chromosomal regions so as to register, signal or perpetuate altered activity states’ [2]. Physically, covalent modifications of histones (the proteins around which DNA is enwrapped, constituting nucleosomes) and methylation of DNA itself are two major biochemical changes strongly influencing how cells, organs, and even individuals make use of their genes over specific periods of time, and under specific environmental constraints. The reason for this is that some of these biochemical marks actually determine the accessibility of genes to RNA polymerase II and to relevant transcription factors. If we could identify if, when and how a given epigenetic mark, or a combination of marks, is induced by an injury, and also understand its real biological impact (good or bad) on the clinical outcome of that injury, then promoting or erasing these marks by using drugs - ‘epidrugs’ - could potentially represent a major breakthrough. By its severity, and the wide repercussions on a number of organs, sepsis exemplifies an event where epigenetics could help to reach unmet needs. Just like goal-directed resuscitation, based on pathophysiology and now a source of therapeutic targets [3], epigenetic modifications could very well be the future for care of patients with MODS: epidrugs are coming.

Endothelial cell dysfunction is a hallmark of sepsis. Bomsztyk *et al.* give insights into how epigenetic modifications are involved by using a mouse model of MODS induced by acute lung injury by *Staphylococcus aureus*. In short, they observed at the early time point of 6 h that the decrease in mRNA of key genes for endothelial function was associated with a decrease in histone marks known to be permissive (that is, to facilitate transcription). As a comparison, repressive marks were not induced at this time point. This suggests that an early intervention preserving or restoring these epigenetic marks would help to preserve endothelial integrity during MODS.

In our opinion, however, three issues need to be addressed to ensure the validity of this promising approach. The first one is technical but of utmost importance: which cell subtype should be studied when considering the epigenome? In homeostasis, many cells coexist in every organ, each with a similar genome but with its own epigenome: it is the very definition of epigenetics. In sepsis, there is a massive infiltration of immune cells in target organs, which makes a cell-specific approach (as opposed to an organ-specific one) even more essential. This is exemplified by the Neutrophil gelatinase-asssociated lipocalin (NGAL), a well-known marker of acute kidney injury, yet abundantly produced by neutrophils [4]. Here, NGAL is shown to be up-regulated in the three organs studied, namely the kidney, the lung, and the liver, but with different epigenetic patterns: permissive acetylation of lysine 4 and 9 of histone H3 is increased in exon 1 in the liver and lung, but not in the kidney. Conversely, repressive methylation of lysine 27 of histone 3 decreases in the lung while increasing in the kidney. This emphasizes the need to integrate the different epigenetic marks in order to not only understand the activity of polymerase 2 in this region of the genome, but also to elucidate which cell acquires or loses these marks. Short of a cell-specific approach, results are complex and potentially confusing. One could speculate that the NGAL gene is differentially regulated in renal and inflammatory cells. We encountered similar problems and recently proposed *ex vivo* cell sorting to circumvent this issue in the kidney, at least in experimental conditions [5].

The second point refers to the control arm: mice undergo neither anesthesia nor mechanical ventilation. The authors argue that a previous microarray did not show substantial decrease in genes of interest, but this does not imply that the epigenome is stable. In addition, anesthesia itself - here isoflurane - has been shown to modify histone marks [6]. We would like to see evidence that, at least for some genes, polymerase 2 is active in endothelial cells; the authors only show negative marks (a decrease in permissive histone modifications, with a decrease in RNA polymerase II density). As explained above, choosing NGAL as a positive control takes the focus away from the endothelium and is still compatible with vascular rarefaction. A ‘positive’ control induced by MODS in endothelial cells is required [7–9].

Finally, how to articulate the cause of injury, the studied phenotype (here, endothelial dysfunction), and epigenetic marks is a general challenge in this emerging field of medicine. This necessitates analysis of the chronology and biological mechanisms whereby epigenetic marks are being bound. Cell metabolism, cell cross-talk, cytokines, and pathogen motifs may all be at issue. Bomsztyk and coworkers have made an important contribution by showing evidence of differences in RNA polymerase II activity. Upstream, cell-oriented mechanisms during sepsis must be clarified if we want to devise new therapies at given time points. We envisage that the impaired energy metabolism observed in sepsis [10] could drive many epigenetic changes. Knowing the highly dynamic nature of epigenetics, with some marks being furtive and others durable, timing of observation surely plays a critical role in how we interpret data; hence the need to be cautious about the causality of pathological changes that might only be temporary. The functional impact of one specific mark is still uncertain.

## Conclusion

Epigenetic studies of systemic diseases such as sepsis-related MODS may help to understand how cell damage proceeds. Obviously, experimental models are essential to explore causality. However, precision regarding the cell subtype involved in epigenetic changes is mandatory, and the timing of analysis is crucial to determine when epidrugs could be incremented in the clinic.

## Abbreviations

MODS: Multiple organ dysfunction syndrome
NGAL: Neutrophil gelatinase-associated lipocalin
